# Changes in type VI collagen degradation reflect clinical response to treatment in rheumatoid arthritis patients treated with tocilizumab

**DOI:** 10.1186/s13075-023-03242-0

**Published:** 2024-01-02

**Authors:** Christian S. Thudium, Peder Frederiksen, Morten A. Karsdal, Anne-Christine Bay-Jensen

**Affiliations:** Nordic Bioscience, Herlev Hovedgade 205-207, Herlev, 2730 Denmark

## Abstract

**Objectives:**

Rheumatoid arthritis (RA) is a chronic autoimmune disease characterized by inflammation in multiple articular joints, causing pain, joint damage, and loss of joint function. Despite the successful development of disease-modifying therapies, the heterogeneity of RA means that a significant proportion of patients respond poorly to treatment. This highlights the need for personalized medicine and predictive biomarkers to optimize treatment efficacy, safety, and cost. This study aimed to explore the relationship between type VI collagen (Col VI) remodeling and clinical response to anti-IL-6 receptor treatment.

**Methods:**

Type VI collagen degradation was quantified using the C6M biomarker, a fragment of type VI collagen degraded by MMPs. Longitudinal differences in average biomarker levels between placebo and treatment groups were estimated using linear mixed models. The predictive capacity of the marker based on change from baseline to 4 weeks was analyzed using logistic regression.

**Results:**

Both 4 mg and 8 mg doses of Tocilizumab (TCZ) reduced serum C6M concentrations compared to the placebo. Furthermore, C6M levels were more reduced in patients responding to treatment compared to early non-responders. A lower early reduction in C6M was associated with reduced odds of ACR treatment response and lowered disease activity.

**Conclusion:**

These findings suggest that quantifying type VI collagen turnover may aid in identifying patients less likely to respond to treatment, indicating a new path towards optimizing patient care. Further studies are needed to validate these findings and explore the underlying mechanisms driving the observed relationships.

**Supplementary Information:**

The online version contains supplementary material available at 10.1186/s13075-023-03242-0.

## Introduction

Rheumatoid arthritis is a chronic autoimmune disease characterized by inflammation of multiple articular joints which manifests as swelling and tenderness, leading to pain and joint deterioration. The symptoms and joint destruction ultimately lead to impaired function and disability in patients. Several disease-modifying biological agents have been successfully developed for RA, which have significantly improved the treatment. However, the heterogeneity of the disease causes a considerable fraction of patients to not respond to the first treatment offered. One of the major challenges in the RA field is therefore to be able to identify the patients that are going to respond to a given treatment and dose, in order to optimize treatment benefit/efficacy, safety, and cost.

Treatment with the αIL-6 receptor agent tocilizumab (TCZ) results in decreased extracellular matrix (ECM) remodeling. The effect of TCZ on ECM biomarkers has been investigated in a number of RA clinical studies, including LITHE and RADIATE [1–4]. Assessing the dose-dependent differences in ECM biomarker levels between 4 and 8 mg/kg doses shows that tissue turnover is markedly different between the two treatment arms [1], and these differences seem to correspond with significant differences in early response rates of ACR20 [5].

Studies investigating the capacity of combinations of biomarkers of type I and III collagen related to synovial inflammation to predict early response to treatment have found associations between baseline levels and response to treatment in the 4 mg/kg treatment group [6]. Furthermore, studies find that early change in markers of type I, III, and collagen degradation as well as bone remodeling markers CTX-I and osteocalcin were associated with treatment response, but that this association varied between treatment groups [4].

Type VI collagen (Col VI) is a ubiquitously expressed protein, present in most tissues where it exerts different molecular and structural roles dependent on the type of tissue [5, 6]. Type VI collagen is found at the interface between the interstitial and the basement membrane where it connects with a range of other matrix proteins, thereby providing a link between cells and the surrounding connective tissue and contributing to the cellular placement in the extracellular architecture [7–9]. It is also present in the pericellular matrix where it is believed to play a role in cell anchoring and survival and has been shown to interact with cell surface receptors involved in intracellular signaling pathways [10, 11]. Type VI collagen interacts with a range of different ECM proteins such as type I [12], II [13], and IV collagen [7]; fibronectin [8]; decorin [13]; and biglycan [14], emphasizing its important structural role in bridging the link between cell and matrix [8].

In joint tissues, it is found mainly in the pericellular matrix (PCM) of articular chondrocytes, where it is involved in its attachment and integrity of cells [15]. In col VI knockout mice alterations in the cartilage such as decreased stiffness and following chondrocyte swelling have been linked with changes in the PCM [10]. Importantly, alterations in the expression of type VI collagen also led to severe articular cartilage degeneration with age and reductions in musculoskeletal parameters such as bone mineral density and secondary ossification [10]. Furthermore, Col VI has been shown to stimulate chondrocyte proliferation and Col VI fragments have been suggested for expansion of chondrocytes in autologous chondrocyte transplantations [16].

Early studies show that Col VI is increased in the synovial interstitial connective tissue of patients with rheumatoid arthritis as compared to healthy controls in which it is only expressed in the synovial membrane [17]. This finding suggests that collagen VI turnover may be increased in RA patients and that changes in disease stage or activity may be reflected in the degradation profile of type VI collagen.

The autoimmune response and following inflammation in rheumatoid arthritis patients results in the release of pro-inflammatory cytokines and increased expression of proteases, such as MMP 2, 3, 9, 12, and 13 [18, 19]. The activity of these proteases results in the release protease generated ECM fragments that are released into the blood where they can be targeted and quantified. Metabolites of the MMP-mediated degradation of Col VI can be measured in serum by the protein fingerprint biomarker C6M, which targets an MMP-cleaved fragment of the α1 chain. The ubiquitous nature of Col VI has previously provided wide use for C6M in different pathologies dependent on the target diseased tissue and established a proof of concept in high tissue turnover diseases. Type VI collagen metabolites have been associated with disease severity in two rat models of liver fibrosis [20]. Moreover, in a study in COPD patients, a positive correlation was observed between C6M and blood eosinophil score, related to the eosinophil bronchitis type of COPD, suggesting a relationship with inflammation at the interface between the interstitial and basement membranes of the lung [21]. In relation to other joint diseases, C6M have been associated with ankylosing spondylitis, a disease characterized by accelerated tissue turnover and increased tissue formation, confirming an association between high joint tissue turnover and increased release of type VI collagen metabolites [22].

The aim of this study was to investigate the association between type VI collagen remodeling and anti-IL-6 receptor treatment. Further to interrogate the association between changes in C6M and clinical outcomes to improve the understanding of the interplay between tissue remodeling, disease activity, and treatment response.

## Materials and methods

### Study design and serum samples

The LITHE study has previously been thoroughly described elsewhere [1, 23, 24] (ClinicalTrials.gov identifier: NCT00106535). This is a 2-year multicenter, randomized, placebo-controlled, and parallel-group phase III trial in patients with moderate to severe RA with an inadequate response to MTX. The patients received either placebo with MTX alone, or either 4 or 8 mg/kg of the IL-6 receptor antibody tocilizumab given intravenously every 4 weeks together with MTX. The biomarker sub-study of LITHE consisted of serum samples from a 1-year double-blinded treatment study, where 741 patients were randomized 1:1:1 to one of the three different treatment arms: placebo, 4 mg/kg, or 8 mg/kg in combination with a stable dose of MTX (10–25 mg/week).

Patients who failed to respond properly to treatment during the study, assessed as < 20% improvement in swollen joint count and tender joint count at week 16 or later, were given the option to receive blinded rescue therapy between 16 and 28 weeks. Patients who did not respond after three doses of second step rescue therapy discontinued treatment. Patients who received rescue therapy at any point were designated as early non-responders (ENR) for the purpose of this study.

Serum for biomarker research was scheduled in the study protocol from patient providing informed consent, and the biomarker study was therefore a prospective study. Blood was collected in the morning after overnight fasting of > 8 h at baseline, weeks 4, 16, 24, and 52. Samples were stored at −80 °C until measurement.

As the current aim was to assess the effect of Il-6 receptor inhibitor treatment on type VI collagen degradation, only patients who provided a baseline sample and at least one post-dosing sample were included in the study.

The ethics committee at every participating institution approved the study and was carried out in line with the Principles of Good Clinical Practice and the Declaration of Helsinki. All participants in the study gave their written consent. This study was not directed by a steering committee.

### Biomarker measures

The collagen VI degradation marker C6M was measured using a competitive ELISA. The assay measures MMP-generated neoepitope fragments of type VI collagen. The technical performance of the assay has previously been described [25]. In brief, a 96-well streptavidin plate was coated with the appropriate biotinylated synthetic antigen dissolved in assay buffer and incubated 30 min at 20℃. Standard or sample was added to appropriate wells followed by addition of 100 µL HRP-conjugated monoclonal antibody against the target sequence and incubated for 1 h at 20 °C. The microtiter plate was then thoroughly washed and 100 µl tetramethyldbenzinidine (TMB) was added and the plate incubated for 15 min at 20℃ in the dark. All the above incubation steps were done with shaking at 300 rpm. TMB reaction was stopped by the addition of 100 µL stop solution (0.18 M sulfuric acid), and the plate read at 450 nm with 650 nm as reference. The standard curve was plotted using a 4-parametric fit model.

### Statistics

Descriptive statistics were calculated based on treatment groups (Table 1). Correlations between baseline biomarker levels and clinical disease descriptives were analyzed by Spearman correlation. For all further analyses, biomarker data were log-transformed to approximate normal distributions. Differences in biomarker levels between placebo and treatment groups at weeks 4 and 16 and between 4, 16, 24, and 52 were assessed using a linear mixed model with interaction between the two fixed effects treatment and time for timepoints 4 and 16 before introducing rescue treatment. Patient ID was included as a random effect to take correlated measurements within the patient into account and baseline biomarker level was included as a covariate (fixed effect). Comparison between ENR and responders between treatment groups were assessed using a linear mixed model with interaction beween treatment, time, and ENR designation, patient ID was included as random effect. Pairwise comparisons of least-square means (LS means) were performed to test for differences in biomarker levels between treatment groups, time points, and ENR designation, using the Holm adjustment method to control for multiple comparisons. *P*-values < 0.05 were considered statistically significant.

**Table 1:** Patient demographics and clinical variables

	**4 MG/KG + MTX (*N* = 227)**	**8 MG/KG + MTX (*N* = 217)**	**Placebo + MTX (*N* = 232)**	**Total (*N* = 676)**	***P* value**
Age, median (Q1, Q3)	51.0 (43.0, 62.0)	54.0 (48.0, 61.0)	52.5 (43.8, 61.0)	53.0 (45.0, 61.0)	0.031
Sex, *n* (%)					0.176
Female	195 (85.9%)	172 (79.3%)	193 (83.2%)	560 (82.8%)	
Male	32 (14.1%)	45 (20.7%)	39 (16.8%)	116 (17.2%)	
BMI, median (Q1, Q3)	26.4 (23.4, 30.4)	26.3 (23.4, 30.5)	27.3 (22.6, 31.4)	26.6 (23.2, 30.8)	0.755
Disease duration, median (Q1, Q3)	7.8 (3.8, 15.1)	7.5 (2.8, 13.5)	6.7 (2.4, 13.9)	7.4 (2.9, 14.0)	0.177
DAS28-ESR, median (Q1, Q3)	6.5 (5.8, 7.2)	6.5 (5.9, 7.1)	6.5 (5.9, 7.2)	6.5 (5.9, 7.2)	0.723
ERN, median (Q1, Q3)	12.4 (4.8, 23.3)	12.5 (4.4, 23.9)	10.4 (3.8, 21.9)	12.0 (4.3, 23.5)	0.354
JSN, median (Q1, Q3)	6.5 (1.8, 16.6)	6.5 (1.2, 16.6)	5.2 (1.1, 15.0)	6.25 (1.2, 16.0)	0.634
SHP, median (Q1, Q3)	18.8 (8.2, 43.7)	19.6 (5.8, 42.7)	16.2 (5.8, 37.8)	18.1 (6.6, 41.9)	0.404
HAQ, median (Q1, Q3)	1.5 (1.0, 1.9)	1.5 (1.3, 1.9)	1.6 (1.1, 2.0)	1.5 (1.1, 1.9)	0.276
VAS PAIN, median (Q1, Q3)	52.0 (39.0, 67.0)	55.0 (42.0, 72.0)	54.0 (44.0, 72.0)	54.0 (41.0, 71.0)	0.362
C6M ng/ml, median (Q1, Q3)	21.8 (15.6, 30.4)	23.1 (13.9, 33.9)	22.1 (14.7, 33.0)	22.5 (14.7, 32.7)	0.802

Data represents median (IQR) for continuous variables or n with percent for categorical variables

*BMI* Body mass index, *DAS28* Disease activity score in 28 joints, *ERN* Erosion, *ESR* Erythrocyte sedimentation rate, *HAQ* Health assessment questionnaire, *JSN* Joint space narrowing, *SHP* Genant modified total sharp score, *VAS* Visual analog scale

Logistic regression was performed to assess associations between biomarker change and clinical outcomes within the individual treatment groups. Clinical outcomes included early non-response, disease activity score in 28 joints (DAS28) remission, DAS28 reduction, and ACR50 response. Odds ratios and 95% confidence intervals per 2-fold change in the biomarker concentration for baseline predictions and ratio from baseline to follow-up for early change were calculated. Adjustments were made to correct for covariates baseline BMI, sex, and age, and Benjamin-Hochberg adjustments were used to correct for false discovery rate (FDR).

The statistical analyses were performed in R version 4.1.0 (R Foundation for Statistical Computing, Vienna, Austria) and RStudio version 1.4.1106 (RStudio, PBC, Boston, Massachusetts). The results were plotted in Graphpad Prism version. 9.1.2 (Graphpad Software, San Diego, California).

## Results

### Patient characteristics in the LITHE biomarker substudy

The full trial description has previously been published by Kremer et al. [26]. A descriptive table of the patient characteristics in the biomarker substudy is provided in Table 1. The biomarker substudy had approximately 220 patients in each arm, with approximately 83% of these being women. The average age was 53 years with a disease duration of approximately 7.4 years. There was no significant difference in DAS, ERN, JSN, SP, HAQ, or VAS PAIN between the treatment groups or between the subgroup and the full study population as previously described by Bay-Jensen et al. [1]. There was no significant difference in C6M levels at baseline between the treatment groups (Table 1).

### Serum C6M modulation as a function of treatment over time

**Fig. 1:**
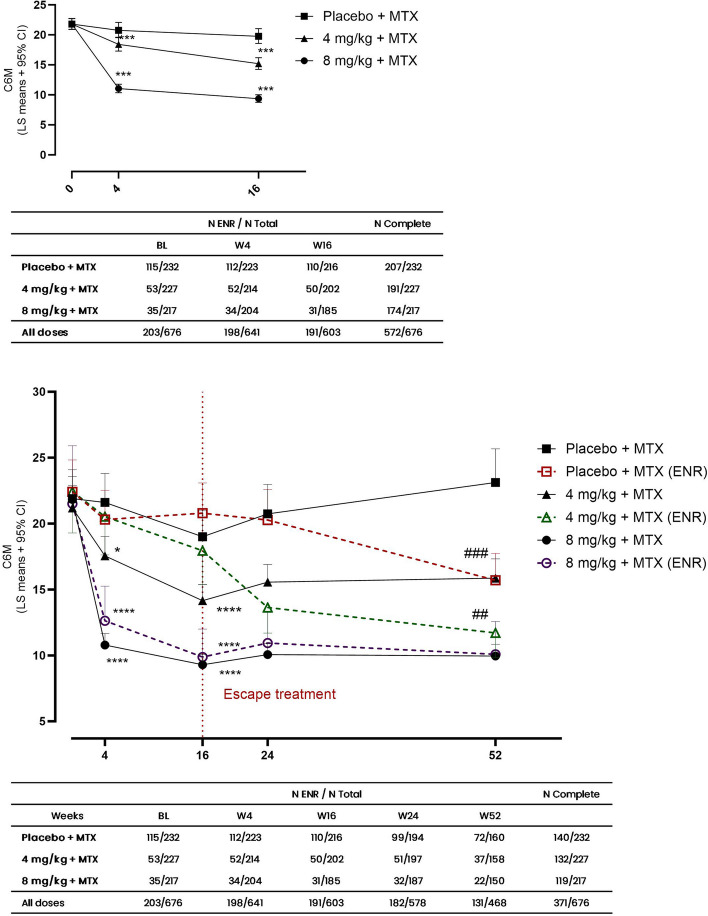
C6M biomarker concentration over time. **A** LS means by treatment and visit for the first 16 weeks in the full population. Treatment and visit as fixed effects and interaction between treatment and visit, baseline C6M as covariate, and patient ID as random effect. **B** LS means by escape status, treatment, and visit. Escape status, treatment, and visit as interacting fixed effects and patient ID as random effect. Tables indicated the number of ENR out of the total number of patients at each timepoint and dose, and the number of patients who provided samples at all timepoints. Data are shown as least square means ± 95% CI and significance levels as * for *p* < 0.05, ** for *p* < 0.01, *** for *p* < 0.001 for comparison to placebo + MTX (**A**) and placebo + MTX responder group (**B**). # designates comparison between ENR and responders within group

To assess the levels of C6M in the three treatment groups the mean estimated serum levels were plotted from baseline to week 16 (Fig. 1A). Data were corrected for baseline concentrations and randomness of the individual patient and interaction between visit and treatment was assumed. Only timepoints up until week 16 were plotted to avoid interference on biomarker levels from potential rescue treatment. C6M levels were reduced in TCZ8 + MTX and TCZ4 + MTX compared to placebo at week 4 (*p* < 0.0001) and week 16 (*p* < 0.0001). The reduction in C6M was a greater in TCZ8 + MTX compared to TCZ4 + MTX at both 4 (*p* < 0.0001) and 16 weeks timepoints (*p* < 0.0001).

Over time C6M levels were reduced to 50% by TCZ8 + MTX at week 4 (CI: 47.4–54.0%, *p* < 0.0001), to 84% by TCZ4 + MTX (CI: 79.3–89.8, *p* = 0.0001) and to 95% by placebo (CI: 89.6–101.2%, *p* = 0.1180), compared to baseline levels (Fig. 1A). After 16 weeks levels were further reduced to 43% of baseline by TCZ8 + MTX (CI: 40.2–45.9%, *p* < 0.0001), to 69% by TCZ4 + MTX (CI: 65.3–74.3%, *p* < 0.0001) and to 90% by placebo (CI: 85.2–96.5%, *p* = 0.0020).

### C6M changes in patients responding treatment

Average levels of C6M were 19% lower in TCZ4 + MTX responder group compared to responders in the placebo + MTX group after 4 weeks (*p* = 0.011) (Fig. 1B). After 16 weeks C6M levels in TCZ4 + MTX dose responding group were 25% lower than responders in the placebo + MTX group (*p* < 0.0001) and 32% lower than ENR in the placebo + MTX group (*p* < 0.0001). There were no differences between the TCZ4 + MTX ENR group and the responder or ENR subgroups in the placebo + MTX group at either 4 or 16 weeks. C6M levels were reduced in the TCZ8 + MTX group compared to both placebo + MTX and TCZ4 + MTX groups at 4 and 16 weeks, irrespective of whether these were responders or ENR group.

After receiving rescue treatment (4 mg/kg) C6M levels in the placebo + MTX ENR group were reduced to approximately the level of the TCZ4 + MTX group responders at week 52. C6M levels in the TCZ4 + MTX ENR group receiving rescue treatment (8 mg/kg) were similarly reduced to close to the level of the TCZ8 + MTX responder and ENR groups.

### Baseline C6M levels and clinical outcomes

We hypothesized that C6M levels at baseline would be associated with the effect of treatment. Baseline C6M levels were tested for association with ENR designation, DAS remission (DAS28 < 2.6), low disease activity (DAS28 < 3.2) or ACR50 response at week 16. The analysis was conducted both globally, controlling for treatment as a covariate along with age, sex, and BMI (Supplementary Table 2), as well as within each treatment group individually (Supplementary Table 3). We found no association between baseline levels of C6M and any of the treatment responses at week 16.

### Early change in C6M and clinical outcomes

We investigated the ability of a 4-week change in C6M to predict clinical treatment response including ENR designation, DAS remission (DAS28 < 2.6), low disease activity (DAS28 < 3.2), and ACR50 after 16 weeks (Table 2). The effect size of the predictor was adjusted for potential confounders and presented as odds ratios reflecting 2-fold change from baseline in C6M.

**Table 2:** Odds ratios for early response to treatment measured by fold change in C6M from baseline to week 4

Response (week 16)	ΔC6M_w4_														
	TCZ4 + MTX					TCZ8 + MTX					Placebo + MTX				
	OR	CI lower	CI upper	*p*	p.adj	OR	CI lower	CI upper	*p*	p.adj	OR	CI lower	CI upper	*p*	p.adj
**Early non-responder**															
Unadjusted	1.27	0.83	1.99	0.279	0.348	1.50	0.99	2.29	0.058	0.115	0.77	0.51	1.13	0.188	0.269
Adjusted	1.28	0.83	2.03	0.267	0.334	1.52	0.99	2.36	0.059	0.117	0.76	0.50	1.13	0.182	0.261
**DAS remission (< 2.6)**															
Unadjusted	0.74	0.40	1.38	0.345	0.383	0.44	0.26	0.70	0.001	0.011	-	-	-	-	-
Adjusted	0.77	0.43	1.38	0.390	0.433	0.41	0.24	0.66	< 0.001	0.005	-	-	-	-	-
**DAS reduction (< 3.2)**															
Unadjusted	0.67	0.41	1.05	0.085	0.141	0.59	0.39	0.86	0.008	0.038	-	-	-	-	-
Adjusted	0.67	0.42	1.06	0.091	0.152	0.52	0.34	0.77	0.002	0.008	-	-	-	-	-
**ACR50**															
Unadjusted	0.62	0.40	0.95	0.031	0.076	0.64	0.43	0.93	0.021	0.071	0.86	0.46	1.63	0.635	0.635
Adjusted	0.62	0.40	0.96	0.035	0.088	0.62	0.42	0.89	0.012	0.041	0.86	0.45	1.63	0.634	0.634

Adjusted for sex, age, and BMI

Odds ratio of response per 2-fold increase in ratio from baseline in C6M, a *p*-value < 0.05 was considered statistically significant

There was no association between the odds of ENR status and change in C6M from baseline to 4 weeks in any of the treatment groups. For DAS28 remission and low disease activity, only fold increase in C6M from baseline in TCZ8 + MTX was associated with reduced treatment response (OR = 0.44, CI: 0.26–0.70 and OR = 0.59, CI: 0.39–0.86, respectively). Associations remained when adjusting for age, sex, and BMI. We found no association with DAS28 remission or DAS28 low disease activity response in TCZ4 + MTX nor in placebo + MTX. Furthermore, fold change in C6M in both TCZ4 + MTX and TCZ8 + MTX were associated with reduced ACR50 response at week 16 (OR = 0.62, CI: 0.40–0.95, and OR = 0.64, CI: 0.44–0.93, respectively). These associations remained significant when adjusting for confounders, but were not significant in the FDR corrected analysis for TCZ4 + MTX.

The long-term suppression in the form of change in C6M levels between baseline and week 16 remained associated with ACR50 after 16 weeks in the TCZ8 + MTX group (OR = 0.63, CI: 0.47, 0.83), while an association was only found in the unadjusted analysis in the TCZ4 + MTX group (OR = 0.73, CI: 0.55–0.97) (Supplementary Table 4). We found no other associations between clinical response to treatment and changes in C6M after 16 weeks in any of the treatment groups.

## Discussion

Aggravated tissue destruction caused by activation of the immune system is a key pathological component of RA. Tissue degradation leads to the release of extracellular matrix degradation fragments released into circulation. In this study, we investigated the potential of the type VI collagen degradation biomarker C6M as a biomarker for prediction of treatment response in patients receiving IL-6R inhibitor treatment.

We found that C6M levels were modulated by treatment, with 4 mg and 8 mg doses having greater reductions than the placebo group at 4 and 16 weeks. We further found that patients who did not have reductions in C6M in the first 16 weeks were more likely to belong to the group in need of rescue treatment. After rescue treatments, introduced at 16 weeks, C6M levels in the placebo group were reduced to those of the 4 mg responder group at 52 weeks, and the 4 mg group receiving rescue treatment dropped to almost the level of the 8 mg group.

C6M is a degradation fragment of type VI collagen release from the proteolytic cleavage by MMPs, and the reduction in C6M, observed here is in line with previous studies showing a general reduction in extracellular matrix degradation fragments and inhibition of tissue turnover with TCZ treatment and other DMOADS [1, 4, 27–29]. Reduction in ECM degradation markers have also been noted in patients treated with baricitinib, underlining the effect that anti-inflammatory treatment has on this type of markers [30]. Despite this, it is worth noting that suppression of C6M in the highest dose, was comparable or greater, compared to similar ECM markers tested in this study (> 50% reduction) [1, 4, 29]. Notably, the C6M marker was also able to separate the two treatment doses from each other, showing a significantly greater inhibitory effect by the highest dose. These findings align with previously published studies showing clear dose-dependent changes in ECM biomarkers related to interstitial and basement membrane degradation following treatment with TCZ [1, 4, 29]. The ability of dose resolution by C6M, which was not possible to achieve by structural assessments in the LITHE study may provide useful evidence when deciding on clinical doses for future anti-inflammatory therapies, as exemplified by the differing Tocilizumab dosing recommendations by EMEA and FDA (www.fda.gov; www.ema.europa.eu/ema).

Lack of early change after 4 weeks in C6M levels was found to be associated with lower odds of achieving clinical response to treatment including DAS28 remission, DAS28 lowering, and ACR50 after 16 weeks. Fold change in C6M levels from baseline in the TCZ8 + MTX group was associated with reduced treatment response for DAS remission and reduction, but not in the TCZ4 + MTX or placebo + MTX group. Fold change in C6M levels in both TCZ4 + MTX and TCZ8 + MTX doses at week 4 were associated with reduced response to treatment for ACR50, even when adjusted for confounders such as age, sex, and disease duration.

Several studies have looked at changes in tissue degradation markers in the context of clinical response. In LITHE, Gudmann et al. previously found that early change after 4 weeks in the MMP-generated type IV collagen biomarker C4M was associated with a higher likelihood of achieving ACR20 response after 16 weeks [29]. Similarly, Bay-Jensen et al. showed that limited effect on tissue degradation markers of type I, II, and III was associated with poorer response to treatment after 16 weeks [1, 4].

In the current study, we found a more limited association between the change from baseline to week 16 and clinical outcomes, and only in the TCZ8 + MTX group for ACR50. We speculate that the longer predictive period effectively suppresses biomarkers in most patients receiving TCZ treatment and therefore the separation between responders and non-responders might even out over time.

In line with these findings, Drobinsky et al. found that an 8-week change in both MMP-generated type III and IV collagen markers was correlated with changes in disease activity score DAS28 at weeks 16 and 24 in the AMBITION, but only in the MTX group, not the TCZ treated group, despite the suppression of biomarkers being more profound in this group. Despite these patients presenting with slightly less severe RA, this study supports that the effective suppression by biological DMOADS, with time, tissue degradation markers will be highly suppressed in most patients.

An interesting observation is that previous studies have found no difference between CRP levels in the 8 mg arm between non-responders and patients responding to treatment in the LITHE cohort, suggesting that in contrast to tissue turnover markers, changes in the acute-phase reactant do not necessarily reflect long term clinical response [1]. Thus, measuring end-products of tissue destruction downstream of inflammatory signals such as cytokines and acute phase reactants may more accurately reflect the total burden and convergence of different pathways rather than single cytokine effects. The patient benefit in response to treatment varies greatly between the range of different RA therapies available, and therefore applying connective tissue biomarkers to identify patient subgroups that may respond optimally to treatment is becoming increasingly important.

Several studies have shown that markers of ECM remodeling even at baseline may provide predictive and prognostic value. Siebuhr et al. found that C1M was prognostic for radiographic progression measured by delta-JSN and modified total sharp score (mTSS) after 24 weeks and 52 weeks in MTX-treated RA patients [3], while Bay-Jensen et. al. showed that C1M combined with the inflammatory marker CRP was prognostic structural progression [31]. Bay-Jensen et al. further showed that a combination of the MMP-generated type III collagen fragment C3M and a metabolite of CRP, CRPM was predictive of ACR50 treatment response to TCZ in the LITHE study [32]. While C6M baseline levels were associated with disease activity scores at baseline (Supplementary Fig. 1) in our study, baseline C6M levels were not predictive of clinical outcomes (Supplementary Tables 2 and 3).

We measured the end-product of tissue degradation in the form of collagen VI using an ELISA targeted against the collagen fragment C6M cleaved off by MMPs. Type VI collagen is a ubiquitously expressed extracellular matrix protein and studies have identified type VI collagen in virtually all connective tissues of the body [5, 33]. In the joint, type VI collagen has mainly been described as the main constituent of the pericellular matrix surrounding articular chondrocytes where it aids in maintaining chondrocyte integrity. Furthermore, older studies have described the presence of type VI collagen deposition in the lining cell layer of both normal and rheumatoid synovium [17, 34]. These early findings also suggest that type VI collagen deposits in the interstitial connective tissue in addition to the lining cell layer in the rheumatoid joint. It is possible that C6M is released from the deteriorating cartilage associated with RA-related inflammation, as well as from synovium, and supports the notion of a role for type VI collagen in the inflamed joint. Given the relatively high correlation with inflammation and clinical outcomes of the joint, it is likely that at least parts of the measured C6M are released from the inflamed synovium. The contribution to the systemic pool of C6M, coming out of the joint, however, is currently unknown.

C6M adds to the pool of extracellular matrix biomarkers reflecting tissue alterations related to RA and in response to anti-inflammatory treatment. Despite the increasing number of markers, these are still confined to exploratory use and have not been validated or qualified according to the setup requirements that are needed for use in diagnostic purposes or as monitoring tools during treatment. Compared to already published biomarkers, of which most are related to collagen degradation, C6M presents with a window of modulation compared to previously well-performing markers, including MMP degraded type I and IV collagen as well as MMP degraded citrullinated vimentin which are reduced by up to 30–50% by TCZ treatment [1, 4, 29].

Several markers have shown predictive ability to identify patients more or less likely to respond. Here, C6M ability to identify treatment responders was most pronounced in the TCZ8 + MTX group, while C6M was only predictive of ACR50 in the uncorrected analysis in the TCZ4 + MTX group. Despite differences in analysis approach limiting direct comparison, C6M seem to be similar or slightly inferior to previous ECM biomarkers such as C1M, C3M, and CRPM in separating treatment responders and non-responders [4].

There are several limitations to the current study which are important to mention. First, this is a phase III clinical study including patients with inadequate response to MTX treatment, and thus the study does not represent the general RA population but only a subset of these. Furthermore, the analysis was performed as a post hoc analysis, and as such, no power calculation was performed, which may likely result in an over-interpretation of the current findings. These findings were only tested in a single clinical study and should be tested in other studies to validate the findings. Future studies should also focus on comparing the predictive ability of C6M with other markers head to head, as well as assess whether a combination of multiple markers together with C6M may provide better prediction.

## Conclusions

The study demonstrates that tocilizumab (TCZ) treatment significantly reduces serum C6M concentrations, a marker of type VI collagen degradation, in rheumatoid arthritis patients. Notably, the reduction was more pronounced in the TCZ8 + MTX group compared to the TCZ4 + MTX group. Patients responding to treatment exhibited a more substantial decrease in C6M levels than non-responders. Early changes in C6M after 4 weeks were indicative of clinical outcomes, including DAS28 in the highest dose and ACR50 in both doses of TCZ. These findings underscore the potential of C6M as a predictive biomarker for treatment efficacy in RA patients. Quantifying C6M levels in patients being treated with anti-inflammatory treatments for RA could be instrumental for personalized treatment strategies in RA, enabling improved clinical outcomes, but further research is warranted to validate and expand upon these insights.

### Supplementary Information

**Additional file 1: Supplementary Figure 1.** Spearman correlations between C6M, clinical characteristics and disease scores. *: *p* < 0.05, **: *p* < 0.01, ***: *p* < 0.001. BMI: Body mass index, DASLERR: Disease activity score in 28 joints (DAS28), ERN: Erosion score, JSN: Joint space narrowing, SHP: Modified total sharp score (mTSS), HAQ: Health assessment questionnaire, VASPAIN: Visual analog score pain.

**Additional file 2: Supplementary Table 1.**

**Additional file 3: Supplementary Table 2.**

**Additional file 4: Supplementary Table 3.**

**Additional file 5: Supplementary Table 4.**

## Data Availability

Data is available upon reasonable request.

## Abbreviations

ACR: American College of Rheumatology
BMI: Body mass index
CRP: C-reactive protein
DAS: Disease activity score
DMARD: Disease-modifying anti-rheumatic drug
ECM: Extracellular matrix
ELISA: Enzyme-linked immunosorbent assay
ERN: Erosion
ESR: Erythrocyte sedimentation rate
EULAR: European Alliance of Associations for Rheumatology
JSN: Joint space narrowing
IL-6: Interleukin-6
MMP: Matrix-metalloproteinase
MTX: Methotrexate
RA: Rheumatoid arthritis
SD: Standard deviation
SHP: Sharp score
TCZ: Tocilizumab
Col VI: Type VI collagen
