# Supplementary material for: Changes in type VI collagen degradation reflect clinical response to treatment in rheumatoid arthritis patients treated with tocilizumab
Source: Arthritis Res Ther. 2024 Jan 2;26:3. doi: 10.1186/s13075-023-03242-0 (PMC10759322; doi:10.1186/s13075-023-03242-0)
Supplement: Supplementary file 1 — Additional file 1: Supplementary Figure 1. Spearman correlations between C6M, clinical characteristics and disease scores. *: p < 0.05, **: p < 0.01, ***: p < 0.001. BMI: Body mass index, DASLERR: Disease activity score in 28 joints (DAS28), ERN: Erosion score, JSN: Joint space narrowing, SHP: Modified total sharp score (mTSS), HAQ: Health assessment questionnaire, VASPAIN: Visual analog score pain. [file 13075_2023_3242_MOESM1_ESM.docx]

Supplementary figure 1


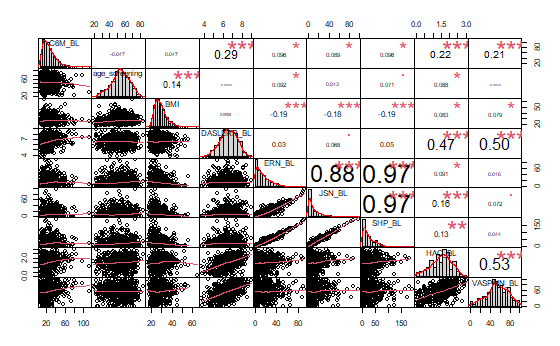


Spearman correlations between C6M, clinical characteristics and disease scores. *: p<0.05, **: p<0.01, ***: p<0.001. BMI: Body mass index, DASLERR: Disease activity score in 28 joints (DAS28), ERN: Erosion score, JSN: Joint space narrowing, SHP: Modified total sharp score (mTSS), HAQ: Health assessment questionnaire, VASPAIN: Visual analog score pain
