# Supplementary material for: Changes in type VI collagen degradation reflect clinical response to treatment in rheumatoid arthritis patients treated with tocilizumab
Source: Arthritis Res Ther. 2024 Jan 2;26:3. doi: 10.1186/s13075-023-03242-0 (PMC10759322; doi:10.1186/s13075-023-03242-0)
Supplement: Supplementary file 2 — Additional file 2: Supplementary Table 1. [file 13075_2023_3242_MOESM2_ESM.docx]

| Week 16 | **4 MG/KG + MTX** | **8 MG/KG + MTX** | **Placebo + MTX** |
| --- | --- | --- | --- |
|  |  |  |  |
| Early non-response | 52/214 | 34/204 | 112/223 |
| DAS remission (<2.6) | 22/201 | 44/182 | 3/215 |
| DAS reduction (<3.2) | 47/201 | 72/182 | 10/215 |
| ACR50 | 58/202 | 60/185 | 24/216 |
