# Supplementary material for: Changes in type VI collagen degradation reflect clinical response to treatment in rheumatoid arthritis patients treated with tocilizumab
Source: Arthritis Res Ther. 2024 Jan 2;26:3. doi: 10.1186/s13075-023-03242-0 (PMC10759322; doi:10.1186/s13075-023-03242-0)
Supplement: Supplementary file 3 — Additional file 3: Supplementary Table 2. [file 13075_2023_3242_MOESM3_ESM.docx]

|  |  | C6M_BL_ | | | | |
| --- | --- | --- | --- | --- | --- | --- |
|  |  | Total | | | | |
| Response Variable (Week 16) | | OR | CI lower | CI upper | p | p.adj |
| **Early Non-responder** | |  |  |  |  |  |
| unadjusted | | 1.05 | 0.91 | 1.21 | 0.521 | 0.917 |
| adjusted | | 1.04 | 0.89 | 1.22 | 0.583 | 0.829 |
| **DAS remission (<2.6)** | |  |  |  |  |  |
| unadjusted | | 1.01 | 0.82 | 1.25 | 0.917 | 0.917 |
| adjusted | | 1.02 | 0.82 | 1.28 | 0.829 | 0.829 |
| **DAS reduction (<3.2)** | |  |  |  |  |  |
| unadjusted | | 0.97 | 0.82 | 1.15 | 0.738 | 0.917 |
| adjusted | | 0.97 | 0.82 | 1.16 | 0.772 | 0.829 |
| **ACR50** |  |  |  |  |  |  |
| unadjusted | | 1.15 | 0.98 | 1.35 | 0.095 | 0.381 |
| adjusted | | 1.16 | 0.98 | 1.37 | 0.082 | 0.329 |
| Adjusted for age, sex, bmi, treatment  OR given a doubling in the predictor; Benjamin-Hochberg was used to correct for FDR; a p-value <0.05 was considered statistically significant | | | | | |  |
|  | | | | | | |
