# Supplementary material for: Changes in type VI collagen degradation reflect clinical response to treatment in rheumatoid arthritis patients treated with tocilizumab
Source: Arthritis Res Ther. 2024 Jan 2;26:3. doi: 10.1186/s13075-023-03242-0 (PMC10759322; doi:10.1186/s13075-023-03242-0)
Supplement: Supplementary file 4 — Additional file 4: Supplementary Table 3. [file 13075_2023_3242_MOESM4_ESM.docx]

|  |  | C6M_BL_ | | | | | | | | | | | | | | |
| --- | --- | --- | --- | --- | --- | --- | --- | --- | --- | --- | --- | --- | --- | --- | --- | --- |
|  |  | 4 mg/kg + MTX | | | | | 8 mg/kg + MTX | | | | | Placebo + MTX | | | | |
| Response Variable (Week 16) | | OR | CI lower | CI upper | p | p.adj | OR | CI lower | CI upper | p | p.adj. | OR | CI lower | CI upper | p | p.adj |
| **Early Non-responder** | |  |  |  |  |  |  |  |  |  |  |  |  |  |  |  |
| unadjusted | | 1.11 | 0.83 | 1.48 | 0.480 | 0.911 | 0.98 | 0.73 | 1.32 | 0.888 | 0.911 | 1.04 | 0.82 | 1.31 | 0.744 | 0.911 |
| adjusted | | 1.08 | 0.81 | 1.45 | 0.602 | 0.861 | 0.98 | 0.72 | 1.32 | 0.878 | 0.890 | 1.05 | 0.83 | 1.33 | 0.700 | 0.861 |
| **DAS remission (<2.6)** | |  |  |  |  |  |  |  |  |  |  |  |  |  |  |  |
| unadjusted | | 0.87 | 0.58 | 1.30 | 0.507 | 0.911 | 1.06 | 0.81 | 1.39 | 0.684 | 0.911 | 1.92 | 0.66 | 6.90 | 0.260 | 0.911 |
| adjusted | | 0.79 | 0.53 | 1.18 | 0.256 | 0.642 | 1.08 | 0.82 | 1.44 | 0.568 | 0.861 | 1.89 | 0.64 | 6.70 | 0.267 | 0.642 |
| **DAS reduction (<3.2)** | |  |  |  |  |  |  |  |  |  |  |  |  |  |  |  |
| unadjusted | | 0.87 | 0.65 | 1.16 | 0.355 | 0.911 | 1.04 | 0.82 | 1.33 | 0.732 | 0.911 | 1.09 | 0.61 | 1.97 | 0.778 | 0.911 |
| adjusted | | 0.84 | 0.62 | 1.12 | 0.232 | 0.642 | 1.05 | 0.82 | 1.35 | 0.712 | 0.861 | 1.12 | 0.62 | 2.04 | 0.717 | 0.861 |
| **ACR50** |  |  |  |  |  |  |  |  |  |  |  |  |  |  |  |  |
| unadjusted | | 1.02 | 0.77 | 1.34 | 0.911 | 0.911 | 1.26 | 0.99 | 1.62 | 0.062 | 0.745 | 1.23 | 0.84 | 1.82 | 0.293 | 0.911 |
| adjusted | | 1.02 | 0.77 | 1.35 | 0.890 | 0.890 | 1.26 | 0.99 | 1.63 | 0.065 | 0.642 | 1.26 | 0.85 | 1.87 | 0.252 | 0.642 |
| Adjusted for age, sex, bmi Adjusted for age, sex, bmi  OR given a doubling in the predictor; ; Benjamin-Hochberg was used to correct for FDR; a p-value <0.05 was considered statistically significant. | | | | | | | | | | | | | | | | |
|  | | | | | | | | | | |  |  |  |  |  |  |
