# Supplementary material for: Changes in type VI collagen degradation reflect clinical response to treatment in rheumatoid arthritis patients treated with tocilizumab
Source: Arthritis Res Ther. 2024 Jan 2;26:3. doi: 10.1186/s13075-023-03242-0 (PMC10759322; doi:10.1186/s13075-023-03242-0)
Supplement: Supplementary file 5 — Additional file 5: Supplementary Table 4. [file 13075_2023_3242_MOESM5_ESM.docx]

|  | ΔC6M_w16_ | | | | | | | | | | | | | | |
| --- | --- | --- | --- | --- | --- | --- | --- | --- | --- | --- | --- | --- | --- | --- | --- |
|  | TCZ4 + MTX | | | | | TCZ8 + MTX | | | | | Placebo + MTX | | | | |
| Response Variable (Week 16) | OR | CI lower | CI upper | p | p.adj | OR | CI lower | CI upper | p | p.adj | OR | CI lower | CI upper | p | p.adj |
| **Early Non-responder** |  |  |  |  |  |  |  |  |  |  |  |  |  |  |  |
| unadjusted | 1.35 | 1.02 | 1.83 | 0.041 | 0.103 | 1.06 | 0.78 | 1.41 | 0.712 | 0.712 | 1.09 | 0.87 | 1.38 | 0.446 | 0.491 |
| adjusted | 1.35 | 1.01 | 1.84 | 0.049 | 0.118 | 1.07 | 0.79 | 1.44 | 0.667 | 0.667 | 1.09 | 0.87 | 1.38 | 0.461 | 0.512 |
| **DAS remission (<2.6)** |  |  |  |  |  |  |  |  |  |  |  |  |  |  |  |
| unadjusted | 0.82 | 0.55 | 1.23 | 0.350 | 0.427 | 0.83 | 0.62 | 1.09 | 0.189 | 0.278 | - | - | - | - | - |
| adjusted | 0.81 | 0.54 | 1.21 | 0.314 | 0.392 | 0.79 | 0.59 | 1.05 | 0.117 | 0.195 | - | - | - | - | - |
| **DAS reduction (<3.2)** |  |  |  |  |  |  |  |  |  |  |  |  |  |  |  |
| unadjusted | 0.76 | 0.56 | 1.01 | 0.064 | 0.117 | 0.85 | 0.67 | 1.08 | 0.202 | 0.278 | - | - | - | - | - |
| adjusted | 0.75 | 0.55 | 1.01 | 0.059 | 0.118 | 0.83 | 0.64 | 1.06 | 0.141 | 0.201 | - | - | - | - | - |
| **ACR50** |  |  |  |  |  |  |  |  |  |  |  |  |  |  |  |
| unadjusted | 0.74 | 0.56 | 0.98 | 0.035 | 0.103 | 0.66 | 0.49 | 0.85 | 0.002 | 0.026 | 0.68 | 0.46 | 0.99 | 0.047 | 0.103 |
| adjusted | 0.73 | 0.55 | 0.97 | 0.032 | 0.106 | 0.63 | 0.47 | 0.83 | 0.001 | 0.015 | 0.64 | 0.43 | 0.95 | 0.028 | 0.106 |
| Adjusted for sex, age, BMI | |  |  |  |  |  |  |  |  |  |  |  |  |  |  |
| Odds ratio of response per 2-fold increase in ratio from baseline in C6M; Benjamin-Hochberg was used to correct for FDR; a p-value <0.05 was considered statistically significant. | | | | | | | | | | | | | | | |
